# Unmasking Spontaneous Coronary Artery Dissection in a Patient With Misleading Symptoms and Initial Imaging Results

**DOI:** 10.1016/j.jaccas.2025.103499

**Published:** 2025-04-23

**Authors:** Judith Gronwald, Johannes T. Kowallick, Andreas Schuster, Alexander Schulz

**Affiliations:** a Department of Cardiology and Pneumology, University Medical Center of Göttingen, Georg-August University, Göttingen, Germany; b FORUM Medizin, Rosdorf, Germany; c School of Biomedical Engineering and Imaging Sciences, King’s College London, London, United Kingdom; d Department of Medicine, Cardiovascular Division, Beth Israel Deaconess Medical Center and Harvard Medical School, Boston, Massachusetts USA

**Keywords:** acute coronary syndrome, cardiac magnetic resonance, myocardial infarction

## Abstract

**Background:**

Spontaneous coronary artery dissection is often missed by conventional, and even invasive diagnostic approaches in patients presenting with acute coronary syndrome.

**Case Summary:**

A 32-year-old man was admitted to the emergency department with acute chest pain after emotional distress. ST-segment elevations and elevated troponin levels were documented. Invasive coronary angiography showed no obstructive coronary artery disease but suspected spasm of the left anterior descending artery. Takotsubo syndrome was considered to be caused by apical wall motion abnormalities on echocardiography. Cardiovascular magnetic resonance imaging revealed ischemic myocardial injury, prompting second-look invasive coronary angiography including optical coherence tomography. A spontaneous coronary artery dissection of the left anterior descending artery with an intramural hematoma and preserved TIMI flow grade 3 was diagnosed, leading to conservative medical management.

**Discussion:**

Although conventional diagnostic assessments may be misleading, multimodal imaging including cardiovascular magnetic resonance imaging helps to accurately identify subentities and mimics of myocardial infarction with nonobstructive coronary arteries, guiding advanced intravascular imaging and therapy.

## Visual Summary

**Figure:**
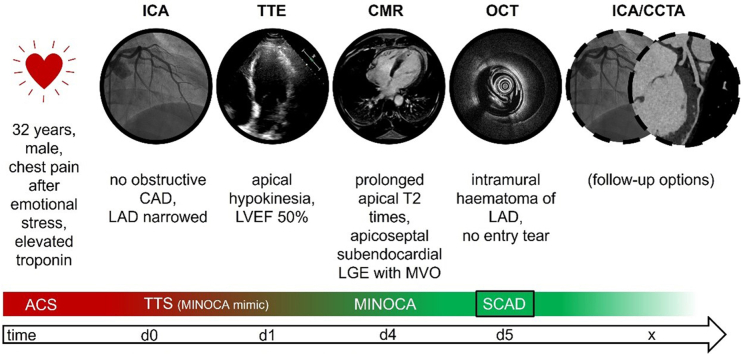

**Visual Summary:**
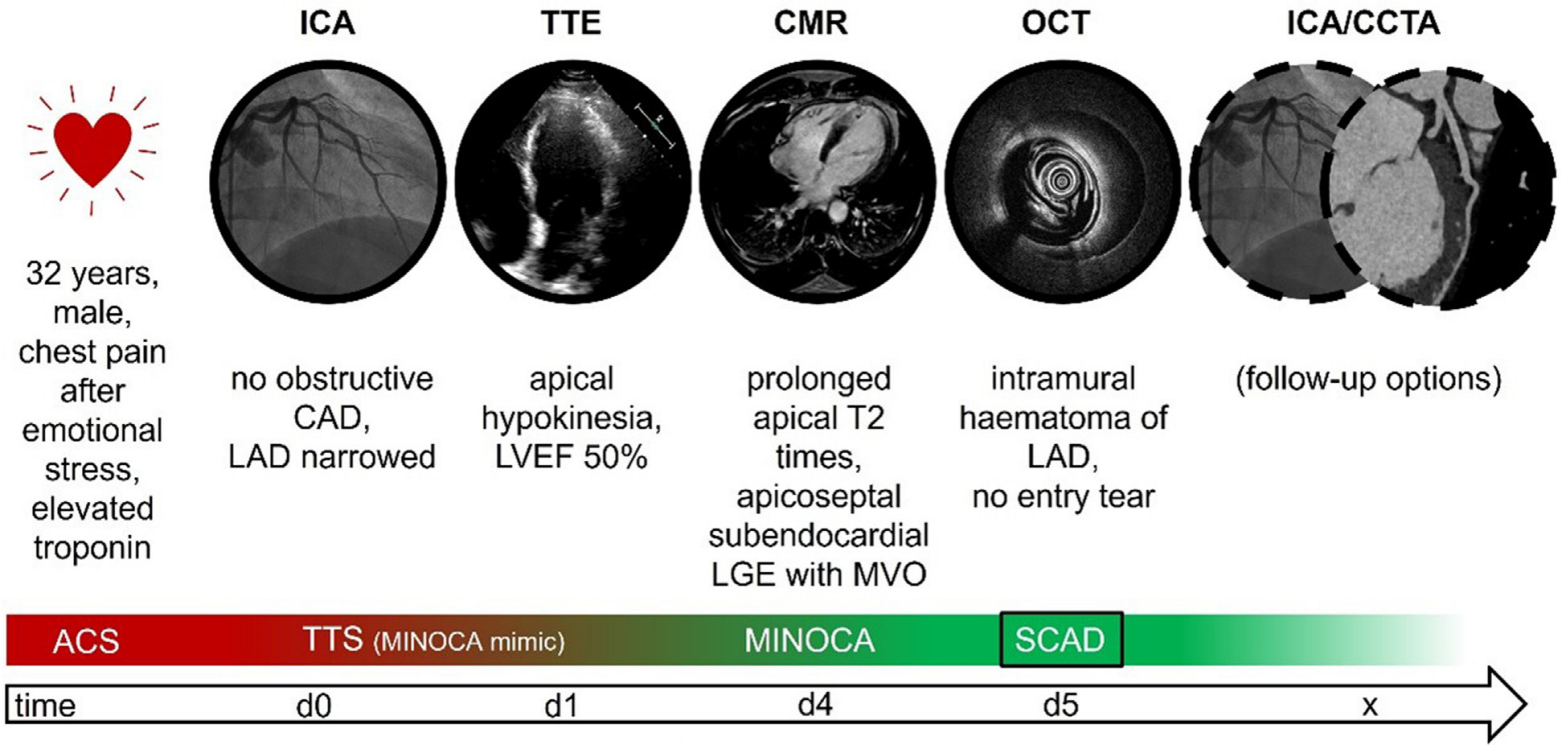
Multimodal Imaging in the Diagnosis and Management of SCAD ACS = acute coronay syndrome; CAD = coronary artery disease; CCTA = cardiac computed tomography angiography; CMR = cardiac magnetic resonance imaging; ICA = invasive coronary angiography; LAD = left anterior descending artery; LGE = late gadolinium enhancement; LVEF = left ventricular ejection fraction; MINOCA = myocardial infarction with nonobstructive coronary arteries; MVO = microvascular obstruction; OCT = optical coherence tomography; SCAD = spontaneous coronary artery dissection; TTE = transthoracic echocordiography; TTS = takotsubo syndrome.

With an estimated prevalence of 4% of patients presenting with acute coronary syndrome (ACS),^1^ spontaneous coronary artery dissection (SCAD) is an important cause of myocardial infarction. SCAD is defined as a wall separation of an epicardial coronary artery by intramural hemorrhage,^2^ either from an intimal tear or through spontaneous hemorrhage arising from the vasa vasorum within the vessel wall. It often occurs in female patients with low prevalence of typical cardiovascular risk factors and can be triggered by extreme physical or emotional stress.^3^ SCAD is classified as a subtype of myocardial infarction with nonobstructive coronary arteries (MINOCA). It is diagnosed in patients presenting with acute myocardial infarction who do not show obstructive coronary arteries on angiography and with no specific alternate diagnosis for the clinical presentation. By definition, the cause of a MINOCA is always ischemic; therefore, nonischemic causes of chest pain with elevated troponin levels, such as myocarditis and takotsubo syndrome (TTS),^4^ are excluded. The latter can imitate the presence of MINOCA and are often referred to as MINOCA mimics. Differentiating between MINOCA subentities and MINOCA mimics often presents a challenge. Young patients in particular often receive an incorrect diagnosis with subsequent consequences for treatment decisions. Initial presentation can often be misleading, especially if symptoms and risk profile are inconsistent. Multimodal imaging, including cardiac magnetic resonance imaging (CMR), may help to avoid misdiagnosis and can be pivotal for clinical management, facilitating therapeutic and interventional decision-making.

**Take-Home Messages**

• In patients presenting with ACS without coronary artery obstruction, early CMR provides guidance for further diagnostic work-up.
• The clinical management of patients presenting with ACS without apparent coronary artery obstruction often remains a challenge within the considerable number of differential diagnoses, especially in patients with misleading symptoms and initial diagnostic results.
• The use of multimodal imaging techniques can provide precise guidance for an accurate diagnosis and individualized decision making for further diagnostic and therapeutic work-up in this context.

## History of Presentation

A 32-year-old male patient with no history of cardiac disease presented to the emergency department with acute chest pain and dyspnea lasting more than 1 hour. The onset of symptoms occurred immediately after attending the funeral of a close friend. Up to this point, there were no preexisting diseases and no cardiovascular risk factors other than class I obesity. Smoking and illicit drug abuse were denied. The patient had consumed a moderate amount of alcohol on the day of admission.

## Investigations

**Figure 1:**
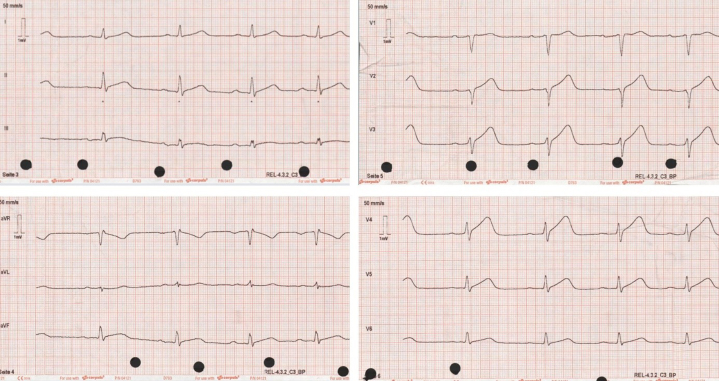
Initial Electrocardiogram The initial electrocardiogram recorded by the emergency physician showed ST-segment elevations in V_2_ to V_5_.

**Figure 2:**
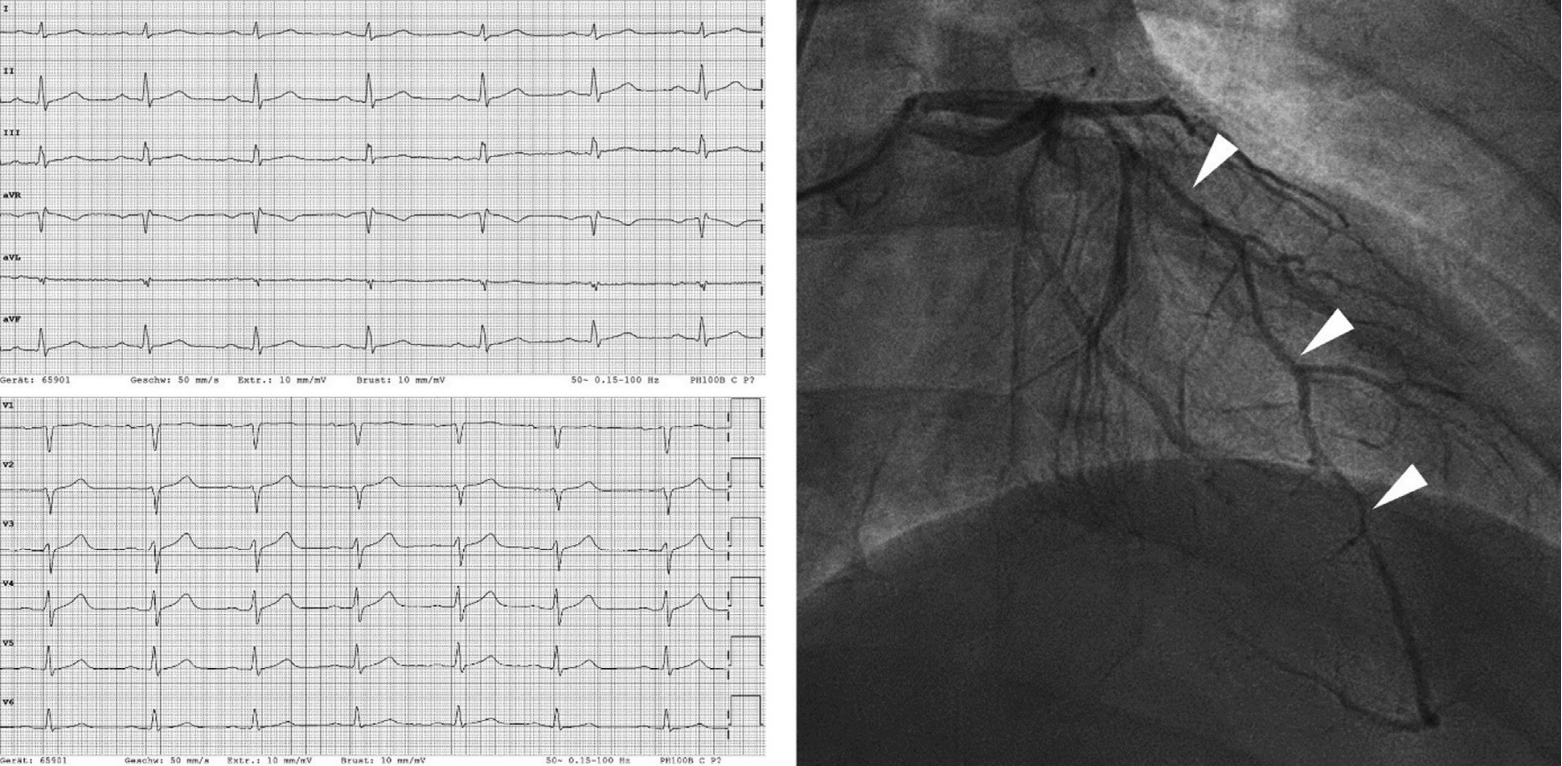
Electrocardiogram at Admission and First Invasive Coronary Angiography (Left) The initial electrocardiogram at the time of admission is shown. (Right) The initial invasive coronary angiography (right anterior oblique 30.0°, cranial 0.0°) showed the absence of obstructed coronary arteries. The left anterior descending artery (white arrowheads) appeared to be narrowed, and given the presence of the extensive radial artery spasm, this finding was interpreted as coronary artery spasm.

**Figure 3:**
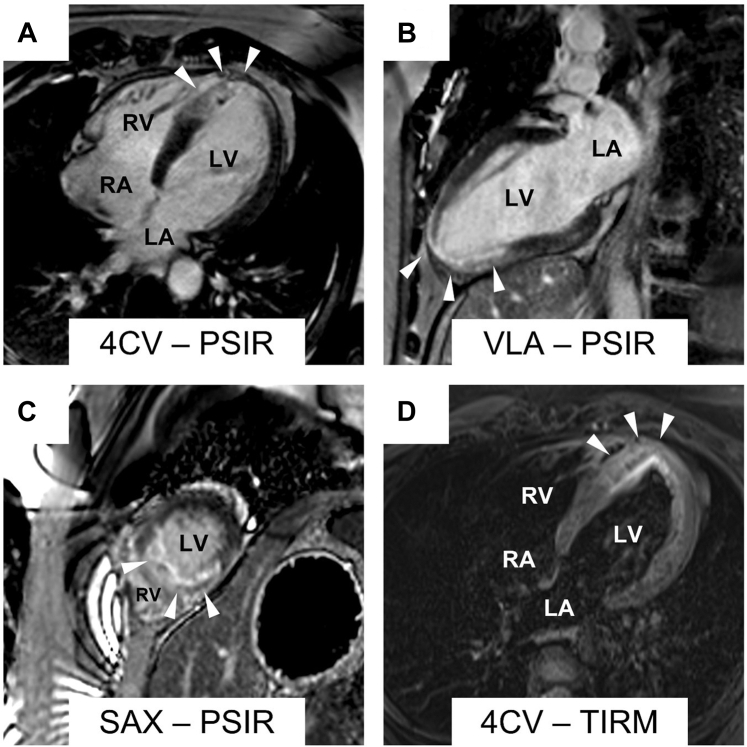
Signs of Ischemic Injury in Cardiac Magnetic Resonance Cardiac magnetic resonance imaging showed signs of subacute myocardial infarction in the territory of the left anterior descending artery. The phase-sensitive inversion-recovery (PSIR) gradient echo sequences (A: four-chamber view [4CV], B: vertical long axis [VLA], C: short axis [SAX]) show late gadolinium enhancement in the apical to midventricular septal region. Turbo-inversion-recovery-magnitude (TIRM) sequences show apical septal and anterior signal enhancement (D). The white arrowheads show signs of subacute myocardial infarction; A to C shows LGE, D shows t2 signal enhancement. LA = left atrium; LV = left ventricle; RA = right atrium; RV = right ventricle.

**Figure 4:**
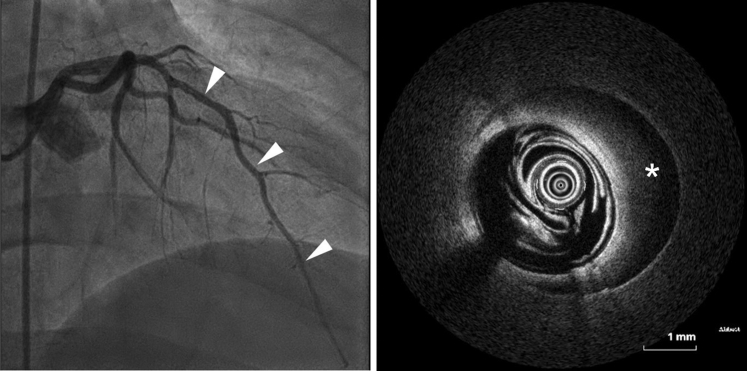
Second Invasive Coronary Angiography With Optical Coherence Tomography The left picture shows the second-look invasive coronary angiography (right anterior oblique 30.0°, CRA 0.0°). The right picture shows the optical coherence tomography of the left anterior descending artery revealing a hematoma (asterisk), leading to the diagnosis of a spontaneous coronary artery dissection. The white arrowheads show the left anterior descending artery, that was narrowed in the first ICA. OCT on the right was performed in this vessel.

An electrocardiogram with ST-segment elevations (V_2_-V_5_) was recorded by the emergency physician (Figure 1). On arrival in the emergency department, the symptoms had resolved, and ST-segment elevations were no longer evident. The patient had stable vital signs (blood pressure 118/65 mm Hg, heart rate 84 beats/min, oxygen saturation 100% on room air) and normal physical examination. Initial laboratory testing revealed elevated troponin I (201 ng/L [≤34.2]) a normal creatine kinase (CK) (179 [30-200 U/L]) but elevated CK-MB (37 U/L [≤25]). After immediate treatment with acetylsalicylic acid and heparin, the patient underwent invasive coronary angiography (ICA) within 1 hour of symptom onset. Initial ICA excluded obstructive coronary artery disease. However, multisegment narrowing of the left anterior descending artery was described and interpreted as vasospasm (Figure 2). No further testing was performed caused by presence of extensive radial artery spasm. Left heart ventriculography showed apical ballooning which led to the working diagnosis of TTS considering remarkable emotional stress. Cardiac enzymes peaked shortly after ICA (troponin I 10,957 ng/L, CK 681 U/L, CK-MB 124 U/L) and then decreased continuously. Transthoracic echocardiography confirmed an apical hypokinesia and a left ventricular ejection fraction of 50%. Within 4 days after symptom onset, CMR was performed for further investigation of the differential diagnosis (Figure 3). CMR showed apicoseptal and anterior signal enhancement in T_2_-weighted sequences, consistent with myocardial edema. Furthermore, increased extracellular volume (34%) and prolonged apicoseptal T_2_ times (53 ms) were observed. Diffuse transmural late gadolinium enhancement (LGE) and microvascular obstruction were seen in concordant myocardial segments. Because pattern and distribution of myocardial injury were compatible with an ischemic cause, a second-look ICA with intravascular imaging was scheduled (Figure 4). Optical coherence tomography (OCT) confirmed the presence of an intramural hematoma (Video 1) resulting in the diagnosis of SCAD.

## Management

As neither an entry nor a thrombus was detected, a conservative approach was proposed in the presence of TIMI (Thrombolysis In Myocardial Infarction) III flow, including medical therapy with dual antiplatelet agents, beta-blockers, angiotensin-converting enzyme inhibitors, and statin. At the time of discharge, the patient was asymptomatic.

## Discussion

Following guideline recommendations, ICA is the undisputed initial diagnostic modality in patients presenting with ACS showing ST-segment elevations or dynamically elevated cardiac troponin.^5^ However, the absence of obstructive coronary artery disease is a challenge for further diagnostic and therapeutic work-up. Although detailed history and echocardiography often point out to a specific cause, they may be misleading. Specifically, the differentiation of ischemic and nonischemic causes imposes challenges in the presence of eg apical hypokinesia because it may indicate both, TTS, or SCAD.

The MINOCA definition has recently been revised and is now restricted to ischemic causes (coronary plaque disease, coronary microvascular dysfunction, coronary vasospasm, coronary thromboembolism, and SCAD). Other nonischemic causes associated with similar clinical presentation and elevated troponin levels, eg, acute myocarditis or TTS, are now classified as MINOCA mimics.^4^

CMR plays a central role in distinguishing the different MINOCA entities from its mimics and has unique capabilities in guiding further diagnostic and interventional steps. Using CMR, nonischemic myocardial injury can be differentiated from ischemic injuries. However, different subentities of ischemic injury, such as plaque rupture or SCAD, may look identical in CMR. Therefore, additional tools such as invasive intravascular imaging are required to determine the definite cause.

Although myocardial edema may be present in nonischemic diseases such as myocarditis or TTS and ischemic diseases such as SCAD, all 3 are characterized by specific patterns. In TTS, the stereotypical pattern of LV dysfunction is complemented by markers for myocardial inflammation and the absence of significant necrosis.^6^ In myocarditis, hyperemia and necrosis are mostly accompanied by a subepicardial pattern of LGE.^7^ Last, in acute myocardial infarction, a subendocardial or transmural pattern of LGE corresponding to a vascular territory is typically seen on CMR.^8^

CMR also provides guidance for subsequent diagnostic procedures including intravascular imaging. Because of its superior resolution, OCT is the preferred imaging modality when evaluating a patient with suspected SCAD by revealing true and false lumens, possible entry tears, and intramural hematoma. Furthermore, it detects plaque rupture and thrombosis.^3^ Although OCT can be a powerful tool, procedural complications have been reported in up to 8% of all patients with SCAD.^9^ Therefore, a careful risk-benefit assessment and a clear indication are desirable before the procedure and may be provided by noninvasive modalities such as CMR. Although MR angiography may also offer a way to visualize coronary artery injury, especially in the proximal coronary segments, limited data on its diagnostic accuracy, particularly for the distal segments, currently constrains clinical application.^10^ Therefore, OCT was chosen as the preferred imaging modality in this case.

With significant improvements in spatial and temporal resolution, cardiac computed tomography angiography (CTA) is a promising noninvasive alternative to ICA for the diagnosis of SCAD.^11^ Combining CMR imaging of myocardial injury with cardiac CTA imaging of the coronary arteries could therefore provide a fully noninvasive diagnostic path, but still needs to be validated in clinical practice. Cardiac CTA could also serve as a follow-up option;^12^ however, further studies on follow-up strategies are needed.

Following the final diagnosis of SCAD, therapy recommendations propose conservative strategies in the acute setting if blood flow is preserved, because percutaneous coronary intervention is associated with worse outcomes and the majority of SCAD heals spontaneously.^13^ Medical treatment options comprise prescription of beta-blockers and antiplatelet therapy. However, caused by the lack of evidence, the use of antiplatelet therapy in particular is controversial,^14^ and further prospective studies are necessary to provide adequate guidelines.

## Conclusions

The diagnosis of SCAD remains a challenge in patients presenting with ACS. Differentiating SCAD from other MINOCA entities and mimics is crucial for further diagnostic and therapeutic steps. CMR presents a reliable and pivotal tool in this process and may guide further diagnostic steps such as OCT or cardiac CTA, which are mandatory to establish final diagnosis of SCAD. Further studies are needed to provide recommendations for therapeutic management and follow-up strategies.

## Funding Support and Author Disclosures

The authors have reported that they have no relationships relevant to the contents of this paper to disclose.

## Appendix

Video 1 Optical coherence tomography of left anterior descending artery.
